# Female genital mutilation and its associated factors among adolescent girls and young women in Tanzania: analysis of the 2022 Tanzania Demographic and Health Survey and Malaria Indicator Survey (2022 TDHS-MIS)

**DOI:** 10.1186/s12889-024-19151-z

**Published:** 2024-07-27

**Authors:** Jovinary Adam, Phinias Charles

**Affiliations:** Jeyibm Investment Tanzania Limited, P.O. Box 20403, Dar Es Salaam, Tanzania

**Keywords:** Female genital mutilation, Adolescent girls, Young women, Prevalence

## Abstract

**Introduction:**

The morbidity and mortality associated with Female Genital Mutilation (FGM) have been clearly documented. Controlling and eventually eradication this practice is very important. Despite a loud call from the WHO and other international organisations, there are extensive nationalities and societies from both developed and developing countries still practising FGM. Understanding the current magnitude and associated factors in Tanzania may bring more light for possible interventions intended to control FGM. This study is timely for this aim.

**Objective:**

To determine the prevalence of female genital mutilation and its associated factors among adolescent girls and young women in Tanzania.

**Methods:**

Secondary data analysis was conducted on data from the 2022 Tanzanian Demographic and Health Survey. The weighted sample included in this study was 2965 adolescent girls and young women aged 15–24 years. Data analysis was performed using Stata 18.0 software. The strength of the association was assessed using the adjusted odds ratio (aOR) along with its corresponding 95% confidence interval (CI).

**Results:**

The overall prevalence of FGM among adolescent girls and young women in Tanzania was 4.9% (95% CI = 3.37, 6.97). The prevalence varied significantly across the zones, ranging from < 1% in both Zanzibar and Southern zones to 19.7% in the Northern zone. Moreover, the results revealed that factors associated with FGM were rural areas (aOR = 2.09, 95% CI = 1.80, 5.44); no education (aOR = 11.59, 95% CI = 4.97, 27.03); poor (aOR = 2.41, 95% CI = 1.20, 4.83); unskilled manuals (aOR = 3.76, 95% CI = 1.97, 7.15); continued FGM (aOR = 3.86, 95% CI = 1.62, 9.18); FGM required by religion (aOR = 8.5, 95% CI = 3.15, 22.96) and watching television at least once a week (aOR = 0.20, 95% CI = 0.70, 1.56) among adolescents and young women in Tanzania.

**Conclusion:**

Female genital mutilation among adolescent girls and young women aged 15–24 years in Tanzania has decreased slightly between 2015/16 and 2022 from 5.9% to 4.9% respectively. This was mostly associated with education level, place of residence, occupation, wealth index, mass media exposure, attitudes towards FGM. More tailored programs focusing on high prevalence zones targeting adolescent girls and young women are needed to end female genital mutilation by 2030.

## Background

Female genital mutilation (FGM) is a significant public health and human rights concern that affects millions of girls and women worldwide. It involves the partial or total removal of external genitalia or other injuries to female genital organs (such as stitching of the labia majora or pricking of the clitoris) for nonmedical reasons [1, 2]. It is estimated that more than 200 million girls and women worldwide are living with the effects of FGM. Of these, 44 million are aged less than 15 years [3]. Moreover, the UNFPA reports that this number is projected to increase to 4.6 million girls by the year 2030 because of population growth and those involved in ending FGM will not intensify their efforts [4]. To date, this practice has been reported in 30 countries in Africa and in a few countries in Asia and the Middle East, with Sub-Saharan Africa leading in female genital mutilation [5].

In East Africa, five countries, Ethiopia, Kenya, Somalia, Uganda and Tanzania, account for almost one-quarter of the global FGM. This means that nearly 50 million girls and young women from this region have undergone FGM [6]. Kenya has the highest (14.8%) FGM prevalence among women between the ages of 15 and 49 followed by Tanzania at 8% [2, 7].

FGM has been widely practiced in Tanzania for years, where it is regarded as a major public health problem. The Tanzania Demographic and Health Survey conducted in 2022 estimated that the prevalence of FGM is 8% among women aged 15–49 years. The prevalence of FGM among women aged 15–49 in Tanzania decreased from 18.0% in 1996 to 8.0% in 2022. Less than 1% of women in Zanzibar have been circumcised, as compared with 9% in Tanzania Mainland. It varies greatly by region whereby Manyara and Arusha are leading with prevalence as high as 43%, and Kigoma, Lindi, Ruvuma, Songwe, Kaskazini Unguja, Kusini Unguja, Kaskazini Pemba and Kusini Pemba with low prevalence of < 1% [2]. The most common type of FGM in Tanzania is type II (cut, flesh removed), with 89% of women undergoing this procedure. Six percent of women underwent a type III procedure (narrowing), and two percent underwent a type I procedure (cut, no flesh removed) [2].

Many communities practice FGM to girls at puberty (9–14 years) and in the reproductive age (15–49 years). These tribes/communities include Iraqw, Waarusha, Maasai, Meru, Sonjo and Somali, Asi, Barabaig, Burunge, Hadza, Rangi, Sandawe and Tiriko, Bena, Hehe, Kinga, Sagara, Chagga from Rombo, Uru and Kibosho, Kikuyu, Hadzabe, Kea (Ndorobo, Mbugwe, Nyaturu, and Pare, Ngoreme; Simbiti; Tatoga; Suba; Zanaki, Kiroba, Kironi, Rieri, Inchugu, Inchage, Wakenye, Warenchoka, Gita and Kabwa, Gogo, Kaguru, Kamba, Nguu, Gogo, and Nguu [8–10].

There are various reasons for FGM practices, which vary across communities in Tanzania. In many societies, FGM is perceived as a means of social control based on the belief that FGM reduces promiscuity as a result of a reduced woman’s libido; to conform and avoid stigma whereby girls/women who are not mutilated are ridiculed, laughed at and excluded/marginalized from communal life; for hygiene, based on the belief that mutilated girls/women are cleaner than those who are not; as rite of passage into womanhood and marriage; as a source of income for female genital mutilators in terms of fees and for parents in terms of bride prices once they marry off their mutilated daughters; to ease childbirth based on a belief that FGM enables a woman to deliver without complications and Cesarean section; and to have control over their husbands [10–12].

The Government of the United Republic of Tanzania is committed towards eliminating all forms of violence against women and children, including FGM. Consequently, the country has developed and is implementing several legal and policy frameworks criminalizing FGM with the aim of eliminating the practice [10]. Moreover, government and anti-FGM stakeholders have been using a number of approaches aimed at changing communities’ perceptions of FGM. These include strengthening the implementation of anti-FGM laws and policies; ensuring local commitment to abandon FGM; media campaigns and advocacy, giving highly needed publicity to the Anti-FGM campaign; the integration of FGM into health services; the creation of awareness among rights stakeholders through youth and children’s clubs; and the strengthening of national and community dialogues on FGM dynamics towards abandoning the practice. These approaches have contributed to increased community awareness, mobilization and advocacy for the prevention and protection of FGM in girls and women, ultimately reducing the FGM incidence rate from 18% in 1996 to 8% in 2022 [2, 10]. The government of Tanzania is committed to achieving sustainable development goals (SDGs), specifically SDG 5, by 2030. Furthermore, in 2018, Ethiopia, Somalia, Uganda, Kenya and Tanzania signed a declaration committing them to support cross-border cooperation and to end female genital mutilation by 2030 [10]. Despite legal and policy restrictions, female genital mutilation is still being practiced in Tanzania.

Several studies reported that education level [13, 14], wealth status [15, 16], place of residence [17], age [9], employment status [18], media exposure [18–20] and region [21] were significantly associated with FGM. Moreover, previous studies conducted in Tanzania focused on female genital amputation in specific areas, such as regions and districts [9, 22, 23]. However, this study uses national and large-scale data set to identify demographic, mass media exposure, geographical, attitude factors. Therefore, this study aimed to determine the prevalence and identify factors associated with female genital mutilation among adolescent girls and young women in Tanzania using the Tanzania Demographic and Health Survey, 2022.

## Methods

### Study setting

The study was conducted in the United Republic of Tanzania in urban and rural areas. It is located in eastern Africa. It is bordered by Kenya and Uganda to the North; Rwanda, Burundi and the Democratic Republic of the Congo to the West; and Zambia, Malawi and Mozambique to the South. The country’s eastern border lies in the Indian Ocean, which has a coastline of 1,424 km. Tanzania has a total area of 945,087 sq. km, including 61,000 sq. km of inland water. The data were collected from 31 geographic regions of the Tanzania mainland and Zanzibar (26 regions of the Tanzania mainland and 5 regions from Zanzibar) [24].

## Data source

Data were extracted from the 2022 Tanzania Demographic and Health Survey, which was conducted jointly by the ICF, Office of the Chief Government Statistician, MoH (Tanzania Mainland and Zanzibar), and National Bureau of Statistics. The survey collected information about female genital mutilation from all women of reproductive age who indicated awareness of FGM. The 2022 Tanzania DHS survey collected data from a nationally representative probability sample of households, women of reproductive age, and men in the sampled households.

## Study design

This secondary study analysed data obtained from the Tanzania Demographic and Health and Malaria Indicator Survey 2022. The study employed a cross-sectional design approach. The sample design was carried out in two stages and intended to provide estimates for the entire country for urban and rural areas. The first stage involves selecting primary sampling units (PSUs) with probabilities proportional to the size within strata, where strata are defined by geographic region and urban/rural areas. The second stage included a systematic sampling of households within the selected clusters.

## Sample size and power of the study

All adolescent girls and young women aged 15–24 years from all selected clusters in Tanzania were considered the study population. The final sample for this analysis included 2965 women aged 15–24 years.

### Data collection tool

A questionnaire was used to collect information from all eligible women aged 15–24 years. The questionnaire was administered to all women aged 15–24 years in the subsample of households selected for the women’s survey.

### Study variables

#### Outcome variable

During the survey, respondent women aged 15–24 years were asked whether they had ever been circumcised prior to the survey, which was measured using the following questions: “Have you yourself ever been circumcised?”. The binary response of respondent circumcised (yes/no) was used as our dependent variable, as was done by previous researchers using DHS data.

#### Exposure variable

**Fig. 1:**
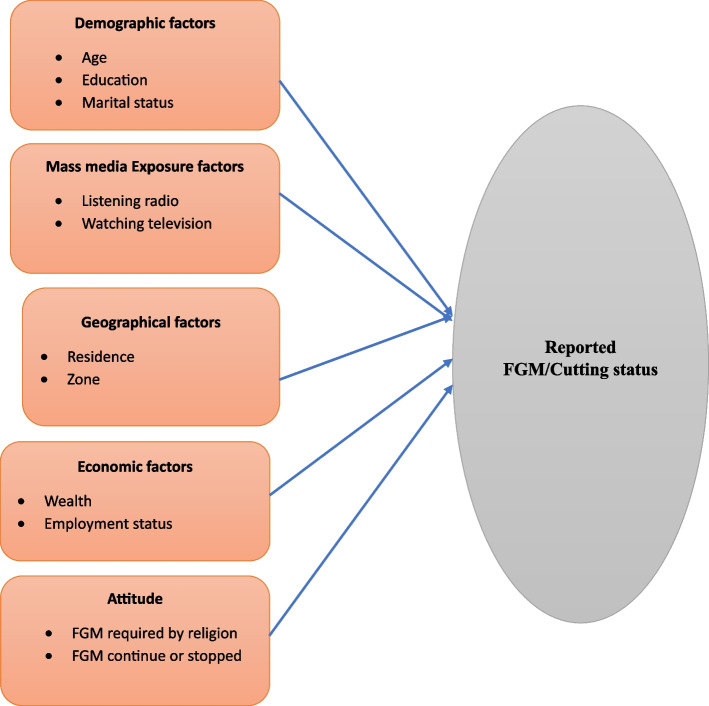
Conceptual framework of the factors associated with FGM among adolescent girls and young women

In this study, “The independent variables selected in this study were categorized into five groups including demographic factors (age, education, marital status); mass media exposure factors (listening radio, watching television); geographical factors (residence, zone); economic factors (wealth, employment status/occupation); and attitude factors (FGM required by religion, FGM continuing or stopping) [18, 21, 25, 26], as demonstrated in Fig. 1 of the conceptual framework. These variables were further categorized into covariates such as age group (15–19 years and 20–24 years); place of residence (urban, rural); education level (higher, secondary, primary, and no education); wealth index (rich, middle, and poor); marital status (never married, married and separated); occupation (not working, professional, agriculture, skilled manual, unskilled manual, other); FGM required by religion (yes, no); FGM continuing or stopped (stopped, continued); listening radio (not at all, less than once a week, at least once a week); and watching television (not at all, less than once a week, at least once a week).

### Statistical data analysis

Data management and analysis were performed using STATA version 18 statistical software (Stata Corp., College Station, Texas, USA). The data were weighted using the sample weights (v005/1000000) as recommended in the DHS data analysis. The data were extracted, recoded and cleaned, and the descriptive results are presented as frequencies and percentages. We examine multicollinearity by variance inflation factor (VIF). The chi-square test was used to examine the association between the outcome variable (female genital mutilation) and categorical independent variables. Variables with p-values less than 0.2 were selected for inclusion in the multivariable binary logistic regression model. Variables with p-values less than 0.05 were considered to be independently significant associated with the FGM. The strength of the association was assessed using the adjusted odds ratio (aOR) along with its corresponding 95% confidence interval (CI).

## Results

### Background characteristics of the study participants

The analysis for this study included a total sample of 2965 adolescent girls and young women aged 15–24 years. The majority of the study respondents were aged 15–19 years 1592 (53.7%). Most of the participants resided in rural areas 1966 (66.3%), and nearly half 1388 (48.0%) had their wealth categorized in the rich quintile. The majority had primary education 1338 (45.1%) and 320 (10.8%) reporting having no formal education. The majority, 1732 (58.4%) reported never married. Other characteristics of the study participants are presented in Table 1.

**Table 1:** Background characteristics and their association with FGM among adolescents and young women in Tanzania (*n* = 2965) (Weighted sample)

Characteristic	Number (%)	Number (%) ever circumcised	χ2; *p*-value
**Age group (years)**			10.6790; 0.032
15–19	1,592 (53.7)	58 (3.7)	
20–24	1,373 (46.3)	86 (6.2)	
**Place of residence**			49.0597; < 0.001
Urban	999 (33.7)	10 (1.0)	
Rural	1,966 (66.3)	134 (6.8)	
**Education**			162.4208; < 0.001
No education	320 (10.8)	57 (17.9)	
Primary	1,338 (45.1)	74 (5.6)	
Secondary and above	1,307 (44.1)	13 (1.0)	
**Wealth**			135.0148; < 0.001
Poor	973 (32.8)	110 (11.3)	
Middle	604 (20.4)	17 (2.8)	
Rich	1,388 (46.8)	17 (1.2)	
**Occupation**			88.3386; < 0.001
Not working	1,600 (53.9)	55 (3.4)	
Professional	308 (10.4)	7 (2.4)	
Agriculture	391 (13.2)	25 (6.3)	
Skilled manual	220 (7.4)	2 (0.8)	
Unskilled manual	373 (12.6)	52 (14)	
Other	73 (2.5)	3 (4.4)	
**Marital status**			68.1616; < 0.001
Never married	1,732 (58.4)	43 (2.5)	
Married	1,102(37.2)	100 (9.1)	
Separated	131 (4.4)	2 (1.2)	
**FGM continue or stopped**			219.7603; < 0.001
Continued	55 (2.2)	28 (51.7)	
Stopped	2,459 (97.8)	116 (4.7)	
**FGM required by religion**			264.0229; < 0.001
No	2,453 (97.6)	111 (4.5)	
Yes	61 (2.4)	33 (53.4)	
**Listening radio**			21.2802; 0.0082
Not at all	1,238 (41.8)	87 (7.0)	
Less than once a week	760 (25.6)	23 (3.1)	
At least once a week	967 (32.6)	34 (3.5)	
**Watching television**			72.7321; < 0.001
Not at all	1,491 (50.3)	121 (8.1)	
Less than once a week	560 (18.9)	16 (2.8)	
At least once a week	914 (30.8)	7 (0.8)	

### The prevalence of FGM among adolescent girls and young women in *Tanzania*

**Fig. 2:**
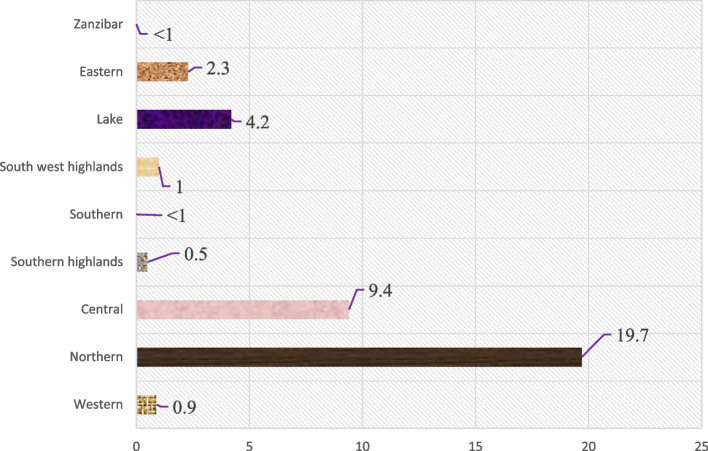
Prevalence of FGM among adolescent girls and young women across geographical zones in Tanzania

The overall prevalence of FGM among adolescent girls and young women in Tanzania was 4.9% (95% CI = 3.37, 6.97). Although the prevalence of FGM among young women 20–24 years was higher, 6.2% (95% CI: 3.98, 9.67), than that of adolescents aged 15–19 years, 3.7% 3.7% (95% CI: 2.41, 5.56), the prevalence was not statistically significant. The prevalence of female genital mutilation among adolescent girls and young women in Tanzania varied significantly across the zones, ranging from < 1% in both Zanzibar and Southern zones to 19.7% in the Northern zone (Fig. 2).

### Factors associated with FGM among adolescent girls and young women in *Tanzania*

As shown in the binary logistic regression output in Table 2, women residing in rural areas had higher odds, (aOR = 2.09, 95% CI = 1.80, 5.44), of experiencing FGM than women residing in urban areas. Women with no formal education had higher odds of being circumcised, (aOR = 11.59, 95% CI = 4.97, 27.03), than those with at least primary education. Moreover, the odds of being circumcised were higher, (aOR = 2.41, 95% CI = 1.20, 4.83), for adolescents and young women in the poor quintile for their counterparts in rich quintile. Unskilled adolescents and young women had significantly higher odds, (aOR = 3.76, 95% CI = 1.97, 7.15), of being circumcised than those not working.

**Table 2:** Logistic regression results on the factors associated FGM among adolescent girls and young women in Tanzania

Variable		OR	95% CI	*P- value*	aOR	95% CI	*P- value*
**Age**	15–19	**Ref**			**Ref**		
	20–24	1.67	0.99–2.80	0.054	0.91	0.51–0.62	0.750
**Place of residence**	Urban	**Ref**			**Ref**		
	Rural	8.57	3.42–21.47	** < 0.001**	2.09	1.80–5.44	**0.034**
**Education**	Secondary and above	**Ref**			**Ref**		
	Primary	7.13	3.61–14.07	** < 0.001**	0.33	0.18–0.61	0.061
	No education	37.10	15.25–90.25	** < 0.001**	11.59	4.97–27.03	** < 0.001**
**Wealth**	Rich	**Ref**			**Ref**		
	Middle	2.58	1.27–5.26	** < 0.001**	0.42	0.20–083	0.138
	Poor	13.85	6.42–29.91	** < 0.001**	2.41	1.20–4.83	**0.013**
**Occupation**	Not working	**Ref**			**Ref**		
	Professional	0.61	0.15–2.46	0.487	0.47	0.86–1.69	0.328
	Agriculture	2.07	1.01–4.23	0.046	1.93	0.34–1.63	0.456
	Skilled manual	0.23	0.05–1.02	0.053	0.24	0.06–1.09	0.065
	Unskilled manual	4.44	2.55–7.73	** < 0.001**	3.76	1.97–7.15	** < 0.001**
	Other	1.21	0.25–5.89	0.817	1.02	0.49–684	0.363
**Marital status**	Never married	**Ref**			**Ref**		
	Married	4.09	2.55–6.59	** < 0.001**	1.37	0.79–2.36	0.260
	Separated	0.52	0.12–2.17	0.367	0.18	0.04–0.77	0.081
**FGM continue or stopped**	Stopped	**Ref**			**Ref**		
	Continue	21.67	10.46–44.87	** < 0.001**	3.86	1.62–9.18	**0.002**
**FGM required by religion**	No	**Ref**			Ref		
	Yes	24.07	11.42–50.71	** < 0.001**	8.50	3.15–22.96	** < 0.001**
**Listening radio**	Not at all	**Ref**			**Ref**		
	Less than once a week	0.45	0.17–0.77	0.009	1.52	0.73–2.84	0.287
	At least once a week	0.33	0.23–0.71	0.002	1.18	0.89–3.03	0.110
**Watching television**	Not at all	**Ref**			**Ref**		
	Less than once a week	0.29	0.14–0.60	** < 0.001**	0.62	0.29–1.32	0.217
	At least once a week	0.08	0.03–0.21	** < 0.001**	0.20	0.70–1.56	**0.002**

Additionally, adolescent girls and young women who needed FGM to continue had higher odds (aOR = 3.86, 95% CI = 1.62, 9.18) of being circumcised than those who needed to stop. The odds of women who said FGM is required by religion were 8.5 times higher (95% CI = 3.15, 22.96), while women who were watching television at least once a week (aOR = 0.20, 95% CI = 0.70, 1.56) had lower odds of being circumcised compared to those who were not watching television at all.

## Discussion

The study assessed the prevalence of female genital mutilation and its associated factors among adolescent girls and young women in Tanzania. The overall prevalence of female genital mutilation among adolescent girls and young women in Tanzania was 4.9%. Our study revealed that the prevalence of FGM among adolescent girls and young women in Tanzania was lower compared to other Sub-Saharan Africa countries such as Sierra Leone [27] (69.8%), Gambia [28] (72.2%), and Ethiopia (52.1%) [29]. The possible reason for the low prevalence in Tanzania might be the existence and enforcement of anti-FGM laws and policies at all levels and the relatively high level of awareness among communities on FGM. Improved awareness has led to openness among traditional leaders, and ex-mutilators now speak openly and act as change agents in their communities. In addition, this might be due to the commitment of some families to end FGM by adopting alternative rites of passage. Hence, FGM cases have been increasingly reported to police stations or at local government authorities and actions taken against perpetrators. This finding is consistent with a study conducted in the Maldives [30] (3.4%), which reported a lower prevalence of FGM among adolescent girls and young women.

The significant factors associated with FGM among adolescent girls and young women in Tanzania include education level, place of residence, occupation, wealth index, mass media exposure, and attitudes towards FGM.

The study findings indicate that adolescent girls and young women in the Northern zone had higher odds of being circumcised compared to other zones. This might be because the northern zone is the home of several ethnic groups, such as Iraqw, Waarusha, Maasai, Chagga, Pare, and Asi, among others, who traditionally practice FGM, and it is rooted in cultural beliefs. Many communities believe that FGM is a necessary rite passage for girls and young women into womanhood and marriage. These findings align with previous studies conducted in Tanzania [23, 31].

Moreover, the odds of being circumcised were 2.1 times higher among adolescent girls and young women who were residing in rural areas compared to those who were residing in urban areas. A possible explanation might be that AGYW in remote areas lack education on the harmful practice of female genital mutilation. Moreover, FGM is often deeply rooted in cultural traditions and beliefs, and these traditions are more strongly upheld and prevalent in rural areas in Tanzania. Our study findings align with those of other studies conducted in Ethiopia and Tanzania [9, 21].

Additionally, the study findings revealed that the odds of being circumcised were higher among uneducated adolescent girls and young women. The possible reason might be that uneducated AGYW are often likely to be influenced by cultural taboos and norms and may possess less autonomy to challenge and question those harmful practices. Due to their low level of education, adolescent girls and young women may not understand the adverse health outcomes associated with practicing female genital mutilation. This finding is consistent with those of studies conducted elsewhere [18, 25].

The results show that adolescent girls and young women from lower socioeconomic backgrounds and those who are unskilled manuals demonstrate a greater inclination towards practices of female genital mutilation. A lower economic status and unskilled manual increase the chance of being circumcised because poverty may make families more likely to adhere to FGM as a way to secure their daughters’ marriage prospects because FGM is seen as a prerequisite for marriage and economic security in some communities. FGM seems to be a source of income for parents in terms of bride prices once they marry off their mutilated daughters, hence leading families to prioritize these cultural norms over the well-being of their daughters. The study findings corroborate other studies conducted in Africa that reported that women are more prone to FGM because of their economic dependency and poverty [26, 32].

Furthermore, adolescent girls and young women who believed that their religion requires FGM and supported FGM to continue had significantly higher odds of being circumcised. The possible reason might be that adolescent girls and young women who have grown in communities with norms and traditions of practicing FGM may feel a strong sense of obligation to uphold these norms even if they are harmful. Another possible reason is that in patriarchy societies such as Tanzania, adolescent girls and young women may have limited autonomy and decision making over their own bodies; hence, some may believe that FGM is necessary to conform to societal norms. The study findings corroborate previous studies conducted in Maldives and Egypt, which indicated that women’s knowledge, attitudes, religion and beliefs are significant predictors of women’s future intentions to practice FGM [33, 34]^.^

Adolescent girls and young women who were exposed to mass media, especially watching television, were less likely to experience female genital mutilation than were those who were not exposed to mass media. Mass media play a significant role in creating awareness about the negative consequences harmful practices including FGM. Adolescent girls and young women exposed to mass media could have a great understanding of healthcare information, including adverse health outcomes associated with practicing female genital mutilation. This implies that they could have less chance of being circumcised. Our findings are consistent with those of other studies conducted elsewhere in Africa [18–20].

## Conclusion

Female genital mutilation among adolescent girls and young women aged 15–24 years in Tanzania has decreased slightly since 2015/2016, from 5.9% to 4.9% in 2022. Factors including education level, place of residence, occupation, wealth index, mass media exposure, and attitudes were associated with FGM practices among adolescent girls and young women. More tailored programs focusing on high prevalence zones targeting adolescent girls and young women are needed to end female genital mutilation by 2030.

## Data Availability

The following information was supplied regarding data availability: 2022 TDHS-MIS report is available at the DHS Program: https://www.dhsprogram.com

## Abbreviations

AGYW: Adolescent Girls and Young Women
aOR: Adjusted Odds Ratio
CI: Confidence Interval
DHS: Demographic and Health Survey
FGM: Female Genital Mutilation
MIS: Malaria Indicator Survey
NBS: National Bureau of Statistics
NIMR: National Institute of Medical Research
TDHS: Tanzania Demographic and Health Survey
UNFPA: United Nations Population Fund
VIF: Variance Inflation Factor
WHO: World Health Organization
